# Comparative Effectiveness of Hysteroscopic Endometrial Resection Versus the 52-mg Levonorgestrel-Releasing Intrauterine System for Heavy Menstrual Bleeding: A Retrospective Cohort Study

**DOI:** 10.3390/medicina62101836

**Published:** 2026-09-23

**Authors:** Zahide Küçük

**Affiliations:** Department of Gynecology and Obstetrics, Dr. Zahide Küçük Practice, Ankara 06530, Turkey; drzahidekucuk@outlook.com or drzahidekucuk@gmail.com; Tel.: +90-532-6290644

**Keywords:** heavy menstrual bleeding, endometrial resection, levonorgestrel-releasing intrauterine system, hysterectomy, sexual function

## Abstract

*Background and Objectives*: To compare the effectiveness, durability, and patient-reported outcomes of hysteroscopic endometrial resection (ER) and the 52 mg levonorgestrel-releasing intrauterine system (LNG-IUS) in women with medically refractory heavy menstrual bleeding (HMB) without structural uterine pathology. *Materials and Methods*: This retrospective cohort study included 242 women treated between January 2021 and December 2024, of whom 117 underwent ER and 125 received a 52 mg LNG-IUS. The primary outcome was hysterectomy during follow-up. Secondary outcomes included bleeding-related symptoms, treatment satisfaction, the change in the Female Sexual Function Index-6 (FSFI-6) total score, LNG-IUS discontinuation/expulsion, and subsequent reintervention. Baseline bleeding severity was characterized using the Pictorial Blood Assessment Chart (PBAC), hemoglobin, and serum ferritin, and abnormal uterine bleeding etiologies were classified according to PALM–COEIN. To address non-randomized treatment allocation, inverse probability of treatment weighting (IPTW) was performed using prespecified baseline covariates. Hysterectomy was further evaluated using Firth penalized logistic regression, Kaplan–Meier analysis, and Cox proportional-hazards models. *Results*: Baseline PBAC, hemoglobin, ferritin, and PALM–COEIN distributions were comparable between groups, whereas age, gravidity, parity, and baseline FSFI differed. After IPTW, all prespecified baseline covariates achieved adequate balance (absolute standardized mean differences < 0.10). Hysterectomy occurred in 3.4% of women after ER and 16.0% after LNG-IUS treatment. In the IPTW-weighted analysis, LNG-IUS was associated with a higher risk of hysterectomy (RR = 8.9, 95% CI 2.8–28.4; *p* < 0.001) and any bleeding-related symptom (RR = 12.8, 95% CI 5.0–32.9; *p* < 0.001), while treatment satisfaction was lower (RR = 0.48, 95% CI 0.39–0.59; *p* < 0.001). Hysterectomy-free survival was lower after LNG-IUS treatment (log-rank *p* < 0.001); the age- and parity-adjusted Cox model showed an HR of 11.6 (95% CI 3.4–39.4; *p* < 0.001), and the IPTW-weighted Cox model showed an HR of 9.8 (95% CI 3.0–32.1; *p* < 0.001). Firth regression similarly demonstrated a higher adjusted odds of hysterectomy with LNG-IUS (aOR = 18.9, 95% CI 5.0–71.9; *p* < 0.001). The 12-month FSFI total score remained lower with LNG-IUS in the IPTW-weighted analysis (B = −2.1, 95% CI −2.5 to −1.6; *p* < 0.001). Among women initially treated with LNG-IUS, 39.2% experienced discontinuation/removal or expulsion and 25.6% underwent subsequent procedural reintervention. *Conclusions*: In this real-world retrospective cohort, ER was associated with fewer subsequent hysterectomies and bleeding-related symptoms, higher treatment satisfaction, and more favorable sexual-function outcomes than the 52 mg LNG-IUS. These associations remained consistent across propensity-weighted, penalized logistic, and time-to-event analyses. Because treatment allocation was non-randomized and preference-based, the findings should be interpreted as comparative associations rather than proof of causal treatment superiority.

## 1. Introduction

Heavy menstrual bleeding (HMB) is a common manifestation of abnormal uterine bleeding (AUB) and an important cause of gynecologic morbidity among women of reproductive and perimenopausal age. In addition to excessive menstrual blood loss, HMB may impair physical, social, occupational, emotional, and sexual well-being and may lead to iron deficiency and iron-deficiency anemia [1,2]. The International Federation of Gynecology and Obstetrics (FIGO) PALM–COEIN system provides a standardized framework for distinguishing structural and non-structural causes of AUB and is particularly relevant when selecting uterus-preserving treatment strategies [3]. Initial management generally includes non-hormonal or hormonal medical therapies according to the clinical characteristics and preferences of the patient [4,5,6].

For women with persistent symptoms despite medical treatment, the 52 mg levonorgestrel-releasing intrauterine system (LNG-IUS) and endometrial resection/ablation represent established uterus-preserving alternatives. The LNG-IUS provides sustained local levonorgestrel delivery and endometrial suppression and is widely used because it is reversible and minimally invasive [7,8]. Nevertheless, persistent or recurrent HMB, irregular bleeding or spotting, pelvic discomfort, device intolerance, and expulsion may result in discontinuation and subsequent intervention [9,10]. Hysteroscopic ER provides a procedural alternative, particularly for women who have completed childbearing and for whom medical treatment has failed or is not preferred [11]. Resectoscopic procedures should be distinguished from non-resectoscopic global ablation techniques because their effectiveness and subsequent intervention profiles may differ. Combined approaches involving endometrial ablation/resection and LNG-IUS have also been investigated [12].

Previous comparative studies and systematic reviews indicate that both LNG-IUS and endometrial ablation/resection can substantially improve HMB-related outcomes, although differences in treatment failure, discontinuation, reintervention, and subsequent hysterectomy have been reported [13,14]. Hysterectomy represents an important definitive surgical endpoint following insufficiently durable uterus-preserving treatment, and time-to-event methods provide additional information by incorporating both the occurrence and timing of hysterectomy [15]. In observational comparisons, however, treatment allocation may be influenced by the patient characteristics, reproductive history, baseline disease burden, clinical counseling, and individual preferences. Propensity-score approaches such as inverse probability of treatment weighting (IPTW) can therefore be useful for improving comparability with respect to measured baseline characteristics [16].

The baseline disease burden and patient-centered outcomes are also relevant when comparing these treatment strategies. Menstrual blood loss can be characterized using the Pictorial Blood Assessment Chart (PBAC), while hemoglobin and serum ferritin provide complementary information regarding the hematologic consequences of chronic blood loss [17,18]. HMB and unpredictable bleeding may also adversely affect sexual activity and sexual well-being. Previous studies evaluating sexual function after LNG-IUS use and operative hysteroscopic treatment have reported variable findings [19,20]. Accordingly, an evaluation of baseline and follow-up sexual function is important when assessing the broader patient-reported outcomes of treatment.

Despite the available evidence, relatively few real-world comparative studies have simultaneously evaluated the baseline bleeding burden, treatment persistence, subsequent reintervention, hysterectomy timing, and sexual-function outcomes while addressing baseline differences between treatment groups. This issue is particularly relevant when definitive outcomes such as hysterectomy are relatively uncommon, because conventional regression estimates may be unstable in the presence of sparse events [21]. Therefore, the present retrospective cohort study compared hysteroscopic ER and the 52 mg LNG-IUS in women with medically refractory HMB without structural uterine pathology. The primary objective was to compare subsequent hysterectomy and hysterectomy-free survival between treatment strategies. Secondary objectives included bleeding-related symptoms, treatment satisfaction, sexual function, LNG-IUS discontinuation or expulsion, and subsequent procedural reintervention. Because treatment allocation was preference-based and non-randomized, adjusted and propensity-weighted analyses were used to account for measured baseline differences.

## 2. Materials and Methods

### 2.1. Study Design and Ethical Approval

This study was designed as a retrospective cohort analysis conducted at the Department of Gynecology, Koru Hospital. Clinical records of women diagnosed with HMB between January 2021 and December 2024 were retrospectively reviewed. Patients who preferred uterine preservation and underwent treatment with either a 52 mg LNG-IUS or hysteroscopic ER were included. All participants had a minimum follow-up period of 12 months after treatment. Treatment selection was based on a clinical evaluation and comprehensive patient counseling, with the final treatment decision determined by patient preference.

The study protocol was reviewed and approved by the Ethics Committee of Koru Hospital (Approval Letter No. 355, dated 3 March 2026) and was conducted in accordance with the principles of the Declaration of Helsinki. Given the non-randomized and preference-based nature of treatment allocation, adjusted analyses were planned to account for baseline differences between the treatment groups. Counseling was provided as part of routine clinical practice by the treating physician and was not based on a formalized study-specific counseling protocol. Therefore, the potential influence of pre-treatment counseling on treatment selection and subsequent patient-reported outcomes was considered when interpreting the findings.

### 2.2. Eligibility Criteria and Patient Selection

Women were eligible for inclusion if they had a diagnosis of HMB, wished to avoid hysterectomy, underwent either LNG-IUS insertion or hysteroscopic ER, had no desire for future fertility, and had follow-up data available for at least 12 months. Patients with incomplete clinical data or an insufficient duration of follow-up were excluded from the study. Among the women excluded because of incomplete clinical data or a follow-up of <12 months (*n* = 23), 9 had incomplete clinical data and 14 had insufficient follow-up. Among those with <12 months of follow-up, 7 had undergone ER and 7 had received the LNG-IUS.

Following the clinical evaluation, patients were informed about the available treatment options, including LNG-IUS insertion and hysteroscopic ER. The advantages, limitations, and potential complications associated with each treatment were explained, and the final treatment decision was made according to patient preference. No randomization or prespecified treatment-allocation algorithm was used. Women who fulfilled the common eligibility criteria were counseled regarding both treatment options, and the clinical suitability for each treatment was considered during counseling. Accordingly, allocation to the ER or LNG-IUS group was determined by the treatment selected by the patient after counseling. Although clinical suitability was considered during counseling, no standardized physician-directed criteria were used to preferentially assign one treatment over the other. Detailed information on all potential determinants of the treatment preference, including the previous hormonal treatment response, contraindications to hormonal therapy, comorbidities, dysmenorrhea, and the preference for amenorrhea versus the preservation of menstruation, was not uniformly available in the retrospective records and therefore could not be incorporated into the treatment-allocation or propensity-score models. Data extraction was based on routinely documented clinical, operative, and follow-up records. Patients were included if they had HMB without an identifiable underlying structural or systemic cause and persistent bleeding despite at least six months of medical treatment, including combined oral contraceptives, progesterone therapy, nonsteroidal anti-inflammatory drugs, or tranexamic acid, as clinically appropriate. HMB was clinically characterized by the need to change pads or tampons every 1–2 h, the passage of large blood clots, bleeding requiring nighttime pad changes, or the presence of iron deficiency or iron-deficiency anemia related to excessive menstrual blood loss.

All patients underwent transvaginal ultrasonography before treatment, and pelvic magnetic resonance imaging was performed when clinically indicated. Patients with uterine fibroids, endometrial polyps, endometrial hyperplasia, adenomyosis, coagulopathy, suspected or confirmed gynecologic malignancy, pregnancy, or a desire for future fertility were excluded. Only patients with a normal uterine cavity and a uterine cavity length of 8–10 cm on transvaginal ultrasonography were included. The complete blood count, coagulation parameters including prothrombin time (PT), international normalized ratio (INR), and activated partial thromboplastin time (aPTT), and beta-human chorionic gonadotropin (β-hCG) testing were obtained before treatment. Diagnostic curettage was performed on all patients, and only those with benign histopathological findings, with endometrial hyperplasia and malignancy excluded, were included in the study.

The patient-selection process, including the eligibility assessment, reasons for exclusion, and final treatment-group distribution, is presented in Figure 1. The final study cohort comprised 242 women, of whom 117 (48.3%) underwent hysteroscopic ER and 125 (51.7%) received the 52 mg LNG-IUS.

### 2.3. Baseline Clinical, Bleeding, and Laboratory Assessment

The baseline demographic and clinical characteristics included age, gravidity, parity, number of vaginal deliveries, and baseline FSFI total score. Baseline menstrual blood loss was assessed using the PBAC, and the baseline hematologic status was characterized using the hemoglobin concentration and serum ferritin level obtained before the index treatment. These variables were extracted from routinely documented pre-treatment clinical and laboratory records. Baseline characteristics were evaluated according to treatment group, and absolute standardized mean differences (SMDs) were used in addition to conventional between-group comparisons to quantify the magnitude of a baseline imbalance.

### 2.4. Classification of Abnormal Uterine Bleeding Etiology

The underlying etiology of AUB was categorized according to the FIGO PALM–COEIN classification using the predominant baseline etiologic category documented during the pre-treatment evaluation. Structural PALM causes were absent because women with endometrial polyps, adenomyosis, uterine leiomyomas, endometrial hyperplasia, or gynecologic malignancy were excluded according to the study eligibility criteria. Women with coagulopathy were also excluded. Among women without structural or systemic causes, AUB-O (ovulatory dysfunction) was assigned when the clinical history indicated irregular or unpredictable menstrual cycles consistent with ovulatory dysfunction. AUB-E (endometrial) was assigned in women with otherwise regular ovulatory cycles and HMB after structural abnormalities, coagulopathy, and other identifiable causes had been excluded, consistent with a primary disorder of endometrial hemostasis. AUB-N (not otherwise classified) was used when the bleeding pattern could not be attributed to the other defined PALM–COEIN categories after completion of the clinical, imaging, laboratory, and histopathological evaluation. Accordingly, the study population consisted of women with non-structural AUB categorized as AUB-O, AUB-E, or AUB-N. The distribution of these etiologic categories was subsequently compared between the treatment groups.

### 2.5. Treatment Procedures

In the surgical group, all hysteroscopic endometrial procedures were performed under direct visualization by the same experienced surgeon to minimize operator-related variability. Procedures were performed using a Karl Storz hysteroscopic system (Karl Storz, Tuttlingen, Germany) in a hospital setting under general anesthesia. Before the procedure, cervical preparation was performed using 200 µg of sublingual misoprostol, and a single prophylactic dose of cefazolin was administered. The procedure was performed using mannitol as the distension medium, a monopolar electrosurgical energy at 80 W, and an intrauterine pressure of 80 mmHg. The mean procedure duration was approximately 20 min. After the procedure, patients were observed for bleeding control and discharged after approximately 6 h if clinically stable. Nonsteroidal anti-inflammatory treatment was prescribed for one week after the procedure. No clinically significant fluid deficit requiring intervention, fluid overload, uterine perforation or rupture, or excessive bleeding was observed.

Technically, the procedure primarily consisted of resectoscopic endometrial resection using a loop electrode, followed by coagulation of the remaining endometrial surface when required. Accordingly, the intervention in the present study should be interpreted as a resectoscopic endometrial resection procedure rather than a non-resectoscopic global ablation technique.

In the LNG-IUS group, insertion was performed after the clinical evaluation and exclusion of pregnancy and suspected endometrial malignancy. Because diagnostic curettage was performed on all patients before treatment, LNG-IUS placement was undertaken only after benign histopathological findings had excluded endometrial hyperplasia and malignancy. The device was not inserted during an episode of uncontrolled active heavy bleeding. In routine clinical practice, both LNG-IUS insertion and hysteroscopic ER were scheduled after the completion of menstrual bleeding and in the absence of active bleeding. Nonsteroidal anti-inflammatory treatment was prescribed for one week after insertion, and the intrauterine position of the LNG-IUS was confirmed at the one-month follow-up visit. No diagnostic curettage, hysteroscopic resection, or other endometrial debulking procedure was performed concurrently with LNG-IUS insertion. Therefore, subsequent bleeding patterns in the LNG-IUS group were not expected to be directly influenced by an immediate concomitant endometrial intervention.

### 2.6. Follow-Up and Outcome Measures

Patients were followed for a minimum of 12 months after treatment. Outcome data were obtained from follow-up visits and a review of medical records. The primary outcome of the study was hysterectomy during the overall follow-up period. The treatment response, bleeding-related symptoms, and patient satisfaction were assessed at the 12-month follow-up, and hemoglobin levels at baseline and at the 12-month follow-up were recorded to evaluate changes in the hematologic status after treatment, while hysterectomy was considered during subsequent clinical follow-up in patients with persistent HMB and an insufficient treatment response. Hysterectomy was considered a clinically relevant downstream outcome after uterus-preserving treatment rather than a uniform marker of treatment failure. No prespecified objective threshold mandated hysterectomy. The decision was individualized according to persistent or recurrent HMB, an insufficient treatment response, the clinical assessment, and the patient preference after counseling. Decisions were predominantly made by the same treating physician.

For women who underwent hysterectomy, the date of the procedure was recorded, allowing the interval between the index treatment and hysterectomy to be determined. Women who did not undergo hysterectomy were followed until their last documented clinical assessment and were censored at that time for time-to-event analyses.

Secondary outcomes included bleeding-related symptoms, treatment satisfaction, and sexual function. Treatment satisfaction was recorded as a binary clinical outcome (satisfied/not satisfied) based on the patient’s documented assessment at the 12-month follow-up and was not measured using a separate validated satisfaction scale. Patients with documentation indicating satisfaction with the treatment were classified as satisfied, whereas those indicating dissatisfaction were classified as not satisfied. During follow-up, LNG-IUS expulsion, patient-requested LNG-IUS removal, hysterectomy due to persistent HMB, and the need for additional treatment were also recorded.

Sexual function was assessed using the 6-item Female Sexual Function Index (FSFI-6), a validated abbreviated version of the original FSFI. The FSFI-6 is a brief questionnaire that evaluates six domains of female sexual function: sexual desire, arousal, lubrication, orgasm, satisfaction, and pain. The FSFI-6 was self-administered by the patients, and the completed questionnaires were reviewed and scored by the same investigator. All included women were sexually active and had complete FSFI-6 data at both baseline and the 12-month follow-up; therefore, the FSFI-6 analyses included all 242 patients (ER, *n* = 117; LNG-IUS, *n* = 125), with no missing FSFI-6 data. The total score is calculated as the sum of the six item scores and ranges from 2 to 30, with higher scores indicating better sexual function. In the original validation study, the FSFI-6 demonstrated good internal consistency (Cronbach’s α = 0.789) and high test–retest reliability (Pearson’s r = 0.95) [22]. Changes in sexual function were assessed by calculating the difference (Δ) between the 12-month and baseline FSFI-6 total scores. The Δ score was defined as the 12-month FSFI-6 score minus the baseline FSFI-6 score. Between-group comparisons were based on the change in the FSFI-6 total score from baseline to 12 months after treatment. Because treatment satisfaction and sexual function are patient-reported outcomes, these endpoints were interpreted cautiously in view of the preference-based treatment allocation.

Any bleeding-related symptom was defined as the presence of either intermenstrual spotting or persistent HMB documented at the 12-month follow-up assessment. These outcomes were retrospectively extracted from routine follow-up records rather than determined using a prospectively standardized bleeding-assessment protocol.

### 2.7. LNG-IUS Discontinuation, Expulsion, and Subsequent Reintervention

Among women initially treated with the 52 mg LNG-IUS, device discontinuation, elective removal, spontaneous complete or partial expulsion, and subsequent procedural reintervention were recorded during follow-up. Primary reasons for LNG-IUS discontinuation/removal or expulsion were classified as persistent or recurrent HMB, persistent intermenstrual spotting or bleeding intolerance, spontaneous complete or partial expulsion, and pain/cramping or other device-related intolerance. These primary reasons were treated as mutually exclusive categories. Subsequent procedural reinterventions, including hysterectomy, hysteroscopic ER, and LNG-IUS replacement or reinsertion, were analyzed separately from device discontinuation and were therefore not summed with discontinuation events. Hysterectomy was considered the definitive reintervention.

### 2.8. Statistical Analysis

Statistical analyses were performed using R software (version 4.5.1; R Foundation for Statistical Computing, Vienna, Austria). Continuous variables were assessed for distributional characteristics and are presented as the mean ± standard deviation (SD) or median [interquartile range (IQR)], as appropriate. Categorical variables are presented as the number and percentage. Between-group comparisons of approximately normally distributed continuous variables were performed using Welch’s independent-samples *t* test, whereas non-normally distributed or ordinal/count variables were compared using the Mann–Whitney U test. Categorical variables were compared using the chi-square test or Fisher’s exact test, as appropriate. All statistical tests were two-sided, and a *p* value < 0.05 was considered statistically significant. There were no missing data for the baseline covariates or the primary and secondary outcome variables included in the principal analyses; therefore, no imputation was performed.

In addition to conventional hypothesis testing, absolute SMDs were calculated to quantify the baseline covariate imbalance between the treatment groups. An absolute SMD < 0.10 was considered indicative of an adequate covariate balance. For the PALM–COEIN classification, the overall distribution of the non-structural AUB categories represented in the cohort (AUB-O, AUB-E, and AUB-N) was compared between treatment groups using the chi-square test.

To address potential confounding associated with the non-randomized treatment allocation, a propensity-score-based IPTW sensitivity analysis was performed. Propensity scores were estimated using multivariable logistic regression with treatment group as the dependent variable and age, gravidity, parity, baseline FSFI total score, baseline PBAC score, hemoglobin, and serum ferritin as prespecified baseline covariates. Stabilized inverse probability weights were subsequently calculated and applied to generate a weighted pseudo-population. The covariate balance before and after weighting was evaluated using absolute SMDs, with values < 0.10 considered indicative of an adequate balance. The distributions of propensity scores and stabilized weights were additionally examined to assess treatment-group overlap, positivity, and the presence of extreme weights. The range and distribution of stabilized weights and the effective sample size (ESS) after weighting were evaluated. No weight truncation or trimming was applied. Robust standard errors were used for IPTW-weighted outcome models. Weighted treatment effects for binary outcomes were expressed as risk ratios (RRs) with 95% confidence intervals (CIs), whereas the treatment effect based on the 12-month FSFI total score was expressed as an unstandardized regression coefficient (B) with a 95% CI. Propensity-score estimation, IPTW, and assessments of covariate balance were performed using the WeightIt and cobalt packages.

Because the primary outcome of hysterectomy occurred in a relatively small number of patients and there was a marked imbalance in event frequency between treatment groups, Firth penalized logistic regression was used to reduce small-sample and separation-related bias. Univariable Firth models were fitted separately for the treatment group, age, and parity. The prespecified multivariable Firth model included the treatment group, age, and parity. Results are reported as odds ratios (ORs) with 95% CIs, with ER used as the reference treatment category. Age was modeled per the 1-year increase and parity per the 1-birth increase. Firth penalized logistic regression analyses were performed using the logistf package.

Time to hysterectomy was defined as the interval from the date of the index treatment (ER or LNG-IUS insertion) to the date of hysterectomy. Patients who did not undergo hysterectomy were censored at their last documented follow-up. Hysterectomy-free survival was estimated using the Kaplan–Meier method, and survival distributions between treatment groups were compared using the log-rank test. Cox proportional-hazards regression was used to estimate hazard ratios (HRs) with 95% CIs, with ER as the reference treatment category. The primary multivariable Cox model was adjusted for age and parity, consistent with the covariate specification used in the primary Firth penalized logistic regression model. As a sensitivity analysis, an IPTW-weighted Cox model was additionally fitted using the stabilized propensity-score weights, with robust standard errors. The proportional-hazards assumption was evaluated using Schoenfeld residuals. Kaplan–Meier and Cox proportional-hazards analyses were performed using the survival package.

For sexual-function outcomes, within-group changes in FSFI scores from baseline to 12 months were assessed using paired analyses appropriate for the distribution of the data, and between-group differences in change scores were evaluated using independent-group comparisons. In addition, the 12-month FSFI total score was evaluated using multivariable linear regression with heteroscedasticity-consistent (HC3) robust standard errors, adjusting for the treatment group, baseline FSFI total score, age, and parity. Model assumptions were evaluated using standard diagnostic procedures. Linearity and homoscedasticity were assessed by an inspection of residual-versus-fitted plots, and residual distributions were evaluated using Q–Q plots. Multicollinearity among covariates was assessed using variance inflation factors (VIFs), with values < 5 considered indicative of no problematic multicollinearity. Regression coefficients (B) with 95% CIs are reported. HC3 robust standard errors were obtained using the sandwich package.

LNG-IUS discontinuation/removal, expulsion, and subsequent reintervention outcomes were summarized descriptively as frequencies and percentages using the entire LNG-IUS group (*n* = 125) as the denominator. Primary reasons for discontinuation/removal or expulsion were treated as mutually exclusive categories. Subsequent procedural reinterventions were analyzed separately from device discontinuation and were therefore not summed with discontinuation events.

All statistical tests were two-sided, and a *p* value < 0.05 was considered statistically significant. Statistical significance was denoted as * *p* < 0.05, ** *p* < 0.01, and *** *p* < 0.001, where applicable.

## 3. Results

### 3.1. Study Population and Baseline Characteristics

A total of 242 women were included in the final analysis, of whom 117 (48.3%) underwent ER and 125 (51.7%) received the 52 mg LNG-IUS. The patient-selection process, including the eligibility assessment and final treatment-group allocation, is presented in Figure 1. The mean age of the overall cohort was 41.6 ± 4.3 years, and 24 women (9.9%) underwent hysterectomy during follow-up. Baseline and follow-up characteristics according to treatment groups are presented in Table 1.

Women undergoing ER were older than those receiving LNG-IUS (43.4 ± 3.0 vs. 39.9 ± 4.7 years; *p* < 0.001; absolute SMD = 0.90). Baseline differences were also observed for gravidity (absolute SMD = 0.30; *p* = 0.029), parity (absolute SMD = 0.40; *p* = 0.012), and the baseline FSFI total score (27.2 ± 2.3 vs. 28.8 ± 2.1; absolute SMD = 0.80; *p* < 0.001). In contrast, the baseline bleeding burden and hematologic parameters were comparable between groups. Median PBAC scores were 286 [244–338] in the ER group and 278 [238–329] in the LNG-IUS group (*p* = 0.472; absolute SMD = 0.09). Mean hemoglobin concentrations were 11.1 ± 1.2 and 11.2 ± 1.1 g/dL, respectively (*p* = 0.503; absolute SMD = 0.09), while median serum ferritin levels were 17.8 [10.6–27.4] and 18.6 [11.2–28.1] ng/mL, respectively (*p* = 0.581; absolute SMD = 0.07). The follow-up duration was comparable between the ER and LNG-IUS groups (16.8 ± 3.7 vs. 16.2 ± 3.5 months; *p* = 0.197), with median follow-up durations of 16.0 [14.0–20.0] months (range, 12–24) and 16.0 [13.0–19.0] months (range, 12–24), respectively.

### 3.2. Distribution of Abnormal Uterine Bleeding Etiologies

The distribution of abnormal uterine bleeding etiologies according to the FIGO PALM–COEIN classification is presented in Table 2. Structural PALM causes were absent because women with polyps, adenomyosis, leiomyomas, endometrial hyperplasia, or gynecologic malignancy were excluded according to the eligibility criteria. AUB-C was likewise absent because women with coagulopathy were excluded.

Among the 242 women, AUB-E was the predominant etiologic category, occurring in 157 (64.9%), followed by AUB-O in 69 (28.5%) and AUB-N in 16 (6.6%). AUB-E was present in 77 women (65.8%) in the ER group and 80 (64.0%) in the LNG-IUS group, whereas AUB-N was identified in 8 women in each group (6.8% and 6.4%, respectively). There was no significant difference in the overall distribution of AUB-O, AUB-E, and AUB-N between the treatment groups (*p* = 0.925), indicating a similar distribution of non-structural AUB etiologies.

### 3.3. Comparative Clinical Outcomes Between Treatment Groups

Clinical outcomes according to treatment strategy are presented in Table 3. Hysterectomy during follow-up occurred in 4 of 117 women (3.4%) following ER compared with 20 of 125 women (16.0%) initially treated with LNG-IUS. This corresponded to an absolute risk difference of 12.6 percentage points. In the crude analysis, the risk of subsequent hysterectomy was 4.7-fold higher in the LNG-IUS group than in the ER group (RR = 4.7, 95% CI 1.6–13.3; *p* = 0.001).

Bleeding-related outcomes also differed between treatment groups. Any bleeding-related symptom at 12 months was documented in 4 women (3.4%) following ER compared with 57 women (45.6%) following LNG-IUS treatment (RR = 13.3, 95% CI 5.0–35.6; *p* < 0.001). Intermenstrual spotting was not documented in the ER group but occurred in 37 women (29.6%) in the LNG-IUS group, corresponding to an absolute risk difference (RD) of 29.6 percentage points (95% CI 21.6–37.6; *p* < 0.001).

Treatment satisfaction was also higher after ER. A total of 110 women (94.0%) in the ER group were classified as satisfied compared with 55 women (44.0%) in the LNG-IUS group (RR for LNG-IUS vs. ER = 0.47; *p* < 0.001) (Table 3).

### 3.4. Sexual Function Outcomes

Changes in the FSFI total score from baseline to 12 months are shown in Table 4. In the ER group, the mean FSFI total score increased from 27.2 ± 2.3 at baseline to 29.2 ± 2.3 at 12 months, corresponding to a mean increase of 2.0 ± 1.0 points (95% CI 1.8–2.2; *p* < 0.001). In contrast, the mean FSFI total score changed from 28.8 ± 2.1 to 28.6 ± 2.3 in the LNG-IUS group, corresponding to a mean change of −0.2 ± 2.2 points (95% CI −0.6 to 0.2; *p* = 0.361).

The between-group difference in the FSFI change was 2.2 points in favor of ER (95% CI 1.7–2.6; *p* < 0.001), with a standardized effect size of Cohen’s *d* = 1.2 (Table 4).

The association between treatment strategy and 12-month FSFI total score was further examined using baseline-adjusted robust linear regression (Table 5). After an adjustment for the baseline FSFI total score, age, and parity, LNG-IUS treatment remained associated with a lower 12-month FSFI total score compared with ER (B = −2.2, 95% CI −2.6 to −1.7; *p* < 0.001). The baseline FSFI total score was positively associated with the 12-month score (B = 0.5 per 1-point increase, 95% CI 0.4–0.6; *p* < 0.001), whereas an increasing age was negatively associated with the outcome (B = −0.2 per year, 95% CI −0.3 to −0.1; *p* < 0.001). Parity was not independently associated with the 12-month FSFI total score (B = 0.0, 95% CI −0.1 to 0.2; *p* = 0.711).

### 3.5. IPTW-Adjusted Treatment Effect Analysis

Given the baseline differences between treatment groups, particularly in age and the baseline FSFI total score, a propensity-score-based IPTW sensitivity analysis was performed. Covariate balance before and after weighting and the corresponding weighted treatment-effect estimates are presented in Table 6.

Before weighting, the absolute SMDs were 0.90 for age, 0.30 for gravidity, 0.40 for parity, and 0.80 for the baseline FSFI total score. Following the application of stabilized IPTW, these values decreased to 0.06, 0.04, 0.05, and 0.07, respectively. The baseline PBAC score, hemoglobin, and serum ferritin also demonstrated adequate post-weighting balance, with absolute SMDs of 0.03, 0.02, and 0.03, respectively. Thus, all prespecified baseline covariates achieved an absolute SMD < 0.10 after weighting. The covariate balance before and after IPTW is additionally illustrated in the Love plot in Figure 2. The propensity-score distributions demonstrated substantial overlap between the treatment groups, with propensity-score ranges of 0.09–0.88 in the ER group and 0.12–0.93 in the LNG-IUS group. The median [IQR] stabilized weights were 0.92 [0.74–1.17] and 0.90 [0.72–1.16], with ranges of 0.55–3.25 and 0.54–3.48, respectively. Stabilized weights >3 were observed in two women (1.7%) in the ER group and three women (2.4%) in the LNG-IUS group, and no stabilized weights exceeded 5. No weight truncation or trimming was applied. The effective sample sizes after weighting were 106.8 and 113.1 in the ER and LNG-IUS groups, respectively. Collectively, these diagnostics supported adequate propensity-score overlap and positivity, without evidence of influential extreme weights.

The IPTW-weighted outcome analyses were consistent with the direction of the primary unweighted comparisons. LNG-IUS treatment was associated with a higher weighted risk of hysterectomy compared with ER (RR = 8.9, 95% CI 2.8–28.4; *p* < 0.001) and a higher risk of any bleeding-related symptom (RR = 12.8, 95% CI 5.0–32.9; *p* < 0.001). Treatment satisfaction remained lower following LNG-IUS treatment (RR = 0.48, 95% CI 0.39–0.59; *p* < 0.001). The weighted analysis of the 12-month FSFI total score likewise remained in favor of ER, with LNG-IUS associated with a 2.1-point lower score (B = −2.1, 95% CI −2.5 to −1.6; *p* < 0.001) (Table 6).

### 3.6. Time to Hysterectomy and Hysterectomy-Free Survival

The times-to-hysterectomy outcomes are presented in Table 7, and Kaplan–Meier hysterectomy-free survival curves are shown in Figure 3. Hysterectomy occurred in 4 women (3.4%) following ER and 20 women (16.0%) following initial LNG-IUS treatment. Among women who experienced the event, the median time to hysterectomy was 14.3 [11.8–16.6] months in the ER group and 10.8 [7.6–13.7] months in the LNG-IUS group.

Estimated hysterectomy-free survival at 12 months was 98.3% in the ER group compared with 91.2% in the LNG-IUS group. At 18 months, the corresponding estimates were 96.5% and 84.2%, respectively. Kaplan–Meier survival distributions differed significantly between the treatment groups (log-rank *p* < 0.001) (Figure 3).

In the unadjusted Cox proportional-hazards analysis, initial LNG-IUS treatment was associated with a higher hazard of subsequent hysterectomy compared with ER (HR = 5.2, 95% CI 1.8–15.2; *p* = 0.003). After an adjustment for age and parity, LNG-IUS treatment remained associated with a higher hysterectomy hazard (adjusted HR = 11.6, 95% CI 3.4–39.4; *p* < 0.001). The IPTW-weighted Cox sensitivity analysis yielded a consistent association (HR = 9.8, 95% CI 3.0–32.1; *p* < 0.001) (Table 7).

### 3.7. Firth Penalized Logistic Regression for Hysterectomy

Because hysterectomy occurred in a relatively small number of women, the association between treatment strategy and hysterectomy was additionally examined using Firth penalized logistic regression. Univariable and multivariable results are presented in Table 8.

In the univariable analysis, LNG-IUS treatment was associated with a higher odds of hysterectomy compared with ER (OR = 5.38, 95% CI 1.85–15.66; *p* = 0.002). An increasing age was also associated with a higher odds of hysterectomy (OR = 1.18 per 1-year increase, 95% CI 1.07–1.30; *p* = 0.001), whereas parity was not significantly associated with the outcome (OR = 1.13 per 1-birth increase, 95% CI 0.84–1.53; *p* = 0.419).

In the multivariable model including the treatment group, age, and parity, LNG-IUS treatment remained independently associated with a higher odds of hysterectomy compared with ER (adjusted OR = 18.9, 95% CI 5.0–71.9; *p* < 0.001). An increasing age was also independently associated with hysterectomy (adjusted OR = 1.5 per 1-year increase, 95% CI 1.3–1.7; *p* < 0.001), whereas parity remained non-significant (adjusted OR = 1.1, 95% CI 0.8–1.5; *p* = 0.661) (Table 8).

### 3.8. LNG-IUS Treatment Outcomes and Reinterventions

LNG-IUS discontinuation and subsequent procedural management are summarized in Table 9. Among the 125 women initially treated with LNG-IUS, 49 (39.2%) experienced device discontinuation/removal or spontaneous expulsion during follow-up. The most frequent reason was persistent or recurrent HMB, resulting in elective removal in 24 women (19.2%). Removal because of persistent intermenstrual spotting or bleeding intolerance occurred in 11 women (8.8%), spontaneous complete or partial expulsion occurred in 8 (6.4%), and pain, cramping, or other device-related intolerance resulted occurred in removal in 6 (4.8%).

Subsequent procedural reintervention following initial LNG-IUS treatment was required in 32 women (25.6%). Hysterectomy was the most frequent definitive reintervention, occurring in 20 women (16.0%). Eight women (6.4%) subsequently underwent hysteroscopic ER, while 4 (3.2%) underwent LNG-IUS replacement or reinsertion. Device discontinuation/removal or expulsion and subsequent procedural reintervention were analyzed as separate outcomes and were therefore not summed (Table 9).

## 4. Discussion

In this retrospective real-world cohort of women with medically refractory HMB, hysteroscopic ER was associated with more favorable clinical and patient-reported outcomes than initial treatment with the 52 mg LNG-IUS. Hysterectomy occurred in 3.4% of women after ER compared with 16.0% after LNG-IUS treatment, while bleeding-related symptoms were substantially less frequent and treatment satisfaction was considerably higher following ER. Sexual-function outcomes showed a similar pattern, with a significant improvement in the FSFI total score after ER but no significant change after LNG-IUS treatment. Importantly, these associations were not confined to crude comparisons. Although the treatment groups differed substantially at baseline in age, gravidity, parity, and the FSFI total score, stabilized IPTW achieved adequate balance across all prespecified covariates, with post-weighting absolute standardized mean differences below 0.10. The principal associations persisted after weighting: LNG-IUS remained associated with a higher risk of hysterectomy and bleeding-related symptoms, lower treatment satisfaction, and a lower 12-month FSFI total score. Time-to-event analyses further demonstrated lower hysterectomy-free survival following initial LNG-IUS treatment, with concordant findings from conventional adjusted and IPTW-weighted Cox models. Firth penalized logistic regression also demonstrated a higher adjusted odds of subsequent hysterectomy after LNG-IUS treatment. Furthermore, 39.2% of women initially treated with LNG-IUS experienced device discontinuation/removal or expulsion, and 25.6% subsequently underwent a procedural reintervention. Although the direction of the principal associations was generally consistent across crude, multivariable, propensity-weighted, penalized logistic, and time-to-event analyses, all of these analyses were derived from the same observational cohort and therefore cannot eliminate residual confounding. Because treatment allocation was non-randomized and preference-based, these findings should be interpreted as comparative associations rather than evidence of causal treatment superiority.

The difference in subsequent surgical treatment observed in our cohort is partly consistent with recent randomized evidence. Huijs et al. reported long-term follow-up of the MIRA randomized trial and found that surgical reinterventions were significantly more frequent after a treatment strategy starting with a 52 mg LNG-IUS than after radiofrequency endometrial ablation, although hysterectomy rates themselves were not significantly different between the groups [23]. Similarly, Beelen et al. found, at 24 months, that surgical reintervention occurred in 27% of women initially treated with LNG-IUS compared with 10% after endometrial ablation [24]. Notably, the 25.6% rate of subsequent procedural reintervention observed among LNG-IUS-treated women in our cohort is close to that reported by Beelen et al., although the specific distribution of subsequent procedures differed. In the present study, 16.0% of women initially treated with LNG-IUS ultimately underwent hysterectomy, 6.4% subsequently underwent hysteroscopic ER, and 3.2% underwent device replacement or reinsertion. Previous comparative studies and systematic reviews have similarly suggested that an initial LNG-IUS strategy may be associated with a greater likelihood of subsequent treatment modification or surgical reintervention than endometrial ablation, although estimates vary according to the follow-up duration and study design [25]. The greater between-group difference in hysterectomy observed in our cohort may reflect the retrospective design, preference-based treatment allocation, differences in patient selection and center-specific thresholds for definitive surgery, and the specific operative technique employed. In particular, our intervention consisted predominantly of direct-visualization resectoscopic endometrial resection followed by coagulation when required, rather than non-resectoscopic radiofrequency ablation. Differences between resectoscopic endometrial resection and non-resectoscopic ablation techniques should therefore be considered when comparing treatment-failure and reintervention rates across studies [4].

The time-to-event findings provide an additional perspective on treatment durability that cannot be captured by cumulative hysterectomy proportions alone. Hysterectomy-free survival remained higher following ER, with estimates of 98.3% versus 91.2% at 12 months and 96.5% versus 84.2% at 18 months. The association persisted in the age- and parity-adjusted Cox model and in the IPTW-weighted Cox sensitivity analysis. Long-term studies of HMB management have demonstrated that escalation to endometrial ablation or hysterectomy may continue for several years after initial conservative treatment, emphasizing that treatment failure is intrinsically time-dependent [26]. Time-to-event approaches are therefore particularly informative when evaluating subsequent surgery because they incorporate both the event occurrence and follow-up time rather than reducing treatment durability to a single binary endpoint. The primary comparison is therefore more appropriately interpreted in terms of the observed absolute event rates: hysterectomy occurred in 3.4% of women following ER and 16.0% following LNG-IUS treatment, corresponding to an absolute risk difference of 12.6 percentage points. Although the Cox, IPTW-weighted Cox, and Firth penalized analyses showed associations in the same direction, only 24 hysterectomy events occurred overall, including four after ER. Consequently, the relatively large HR and OR estimates were accompanied by wide CIs and should be interpreted cautiously. These adjusted relative estimates should therefore be regarded as supportive analyses rather than precise measures of the magnitude of the treatment effect [21].

Bleeding control and patient experience represent another important dimension of the present findings. Any bleeding-related symptom was recorded in 45.6% of women treated with LNG-IUS compared with only 3.4% after ER, and intermenstrual spotting occurred exclusively in the LNG-IUS group. Nevertheless, the effectiveness of LNG-IUS for HMB is well established. Creinin et al. demonstrated a median reduction in menstrual blood loss exceeding 90% within six months after insertion of a 52 mg levonorgestrel intrauterine device, with treatment success in more than 80% of participants [27]. Furthermore, Laporte et al. reported that approximately 91% of LNG-IUS users were satisfied with the method, with a reduction in menstrual bleeding and dysmenorrhea among the main reasons for satisfaction [28]. These findings contrast the 44.0% satisfaction rate observed among LNG-IUS-treated women in our cohort. Previous comparative evidence indicates that both LNG-IUS and endometrial ablation can substantially reduce menstrual bleeding, although ablation may provide greater reductions in bleeding scores in some populations [25]. The comparatively low satisfaction observed in our LNG-IUS group may partly reflect the highly selected nature of the study population, in which women had persistent HMB despite at least six months of previous medical treatment, as well as differences in expectations, counseling, the tolerance of irregular bleeding, and the retrospective binary assessment of treatment satisfaction.

The detailed LNG-IUS treatment-course data provide further context for these findings. Overall, 39.2% of LNG-IUS-treated women experienced device discontinuation/removal or spontaneous expulsion. Persistent or recurrent HMB was the most frequent reason, accounting for 19.2% of the entire LNG-IUS group, while persistent intermenstrual spotting or bleeding intolerance accounted for a further 8.8%. Spontaneous complete or partial expulsion occurred in 6.4%, and pain, cramping, or other device-related intolerance accounted for 4.8%. The discontinuation of LNG-IUS during treatment for HMB is well documented and may occur because of persistent bleeding, irregular bleeding or spotting, pain, adverse effects, expulsion, or patient preference [25]. Altered bleeding patterns are particularly relevant because the patient perception of treatment success depends not only on an absolute reduction in menstrual blood loss but also on bleeding predictability and acceptability. Long-term observational evidence also indicates that the continuation of LNG-IUS declines progressively over time in women initially managed medically for HMB [26].

Sexual function provides an additional patient-centered perspective on the treatment response. In our study, the FSFI total score increased by 2.0 points after ER, whereas no significant improvement was observed after LNG-IUS treatment, and LNG-IUS remained associated with a 2.2-point lower 12-month FSFI total score after baseline adjustment. Abul et al. similarly demonstrated treatment-related differences in sexual function among women with abnormal uterine bleeding, reporting higher total FSFI scores, as well as higher desire, arousal, and lubrication scores, among women receiving cyclic dydrogesterone than among those using LNG-IUS [29]. From a broader perspective, Vitale et al. emphasized that abnormal uterine bleeding may negatively affect sexuality and quality of life and that effective uterus-preserving hysteroscopic treatment can improve patient-centered outcomes. HMB may interfere with sexual activity through several mechanisms, including unpredictable bleeding, concerns regarding bleeding during intercourse, pelvic discomfort, fatigue related to iron deficiency or anemia, and adverse effects on psychological well-being and body image. Improved bleeding control after successful treatment may therefore contribute indirectly to an improvement in sexual function and quality of life [30].

The sexual-function association was also preserved after propensity weighting, with LNG-IUS associated with a 2.1-point lower 12-month FSFI total score after balancing the prespecified baseline covariates. This finding is particularly relevant because the baseline FSFI total score was substantially different between treatment groups before weighting. The concordance between conventional baseline-adjusted regression and IPTW-weighted analysis reduces concern that the observed difference was explained solely by a measured baseline imbalance. Nevertheless, sexual function is multidimensional and may be influenced by age, relationship factors, psychological well-being, hormonal effects, treatment expectations, and changes in bleeding patterns. Studies evaluating sexual function after LNG-IUS have produced heterogeneous findings, with improvement, neutral effects, and treatment-specific differences reported across populations and indications [29]. Moreover, the present study was not designed to establish a minimum clinically important difference specifically for FSFI-6. Accordingly, the observed between-group difference should be interpreted as an adjusted association rather than definitive evidence of a clinically meaningful treatment-specific effect on sexual function.

Another important feature of the present cohort was the characterization of the baseline bleeding burden. Baseline PBAC scores were similar between groups, as were hemoglobin concentrations and serum ferritin levels. Thus, despite marked differences in age and the baseline FSFI, the treatment groups did not appear to differ materially in measured menstrual bleeding burden or its hematologic consequences before treatment. PBAC provides a practical semiquantitative approach to assessing menstrual blood loss and has been widely used as an outcome measure in clinical studies of HMB. Hemoglobin and ferritin provide complementary information regarding the hematologic consequences of chronic excessive menstrual blood loss and iron depletion [17,18]. The comparability of these parameters between groups is therefore important because baseline disease severity could otherwise provide an alternative explanation for subsequent differences in bleeding-related outcomes, satisfaction, or progression to definitive surgery.

The etiologic composition of the cohort also deserves consideration. According to the PALM–COEIN framework, all included women had non-structural AUB because polyps, adenomyosis, leiomyomas, endometrial hyperplasia or malignancy, and coagulopathy were excluded according to the eligibility criteria. AUB-E was the predominant category, followed by AUB-O and AUB-N, and their distribution did not differ between treatment groups. The FIGO PALM–COEIN system provides a standardized framework for distinguishing structural causes of AUB from non-structural etiologies and facilitates consistent investigation and reporting. The etiologic classification is also relevant when interpreting treatment effectiveness because treatment selection and expected outcomes may vary according to the underlying cause of AUB [3]. The relatively homogeneous non-structural composition of the present cohort therefore facilitates an interpretation of the treatment comparison but simultaneously limits generalizability to women with fibroids, adenomyosis, polyps, or other structural causes of HMB.

The relationship between age and subsequent hysterectomy in our adjusted analysis deserves particular consideration. Increasing age was independently associated with a higher odds of hysterectomy in the overall cohort, whereas parity was not. This finding differs from several ablation-specific studies. In a population-based analysis of 76,446 women, McGee et al. reported that an advancing age at the time of endometrial ablation was associated with a lower probability of subsequent hysterectomy, with the cumulative hysterectomy rate continuing to increase over long-term follow-up [31]. Similarly, Meijer et al. reported an 11.4% hysterectomy rate after NovaSure ablation and identified factors such as a higher BMI and larger uterine cavity dimensions as predictors of subsequent hysterectomy [32]. The difference in the direction of the age association may arise because our regression model evaluated the entire cohort across two different treatment pathways rather than predictors of failure among ablation patients alone. Age may also influence the treatment selection, reproductive priorities, proximity to menopause, tolerance of persistent symptoms, and the threshold for proceeding to definitive surgery. Moreover, age was markedly imbalanced between our groups and is closely linked to clinical decision-making. Accordingly, the age association observed in our study should not be interpreted as evidence that an increasing age is a biological predictor of resectoscopic endometrial resection failure.

The substantial baseline differences between treatment groups highlight a central methodological challenge inherent to real-world comparative effectiveness research. Women undergoing ER were older and differed from LNG-IUS-treated women in gravidity, parity, and baseline FSFI total scores. Such differences may arise when treatment allocation reflects clinical counseling and patient preference rather than random assignment, making confounding by indication an important consideration in observational comparisons of medical and surgical treatment pathways. We used stabilized IPTW as a sensitivity analysis to improve comparability across measured baseline characteristics. All prespecified covariates achieved an absolute SMD below 0.10 after weighting, and the principal treatment associations remained directionally consistent. An absolute SMD below 0.10 is commonly used as an indicator of adequate covariate balance in propensity-score analyses [16]. The principal associations remained directionally consistent after weighting, suggesting that the observed differences were not explained solely by the measured baseline imbalances. However, propensity-score methods can balance only observed covariates and cannot eliminate residual confounding from unmeasured factors.

From a clinical perspective, the present findings support individualized counseling rather than a simple hierarchy of treatment superiority. Deehan et al. concluded, in an updated systematic review, that endometrial ablation/resection is an effective uterus-preserving alternative to hysterectomy, although hysterectomy provides more definitive bleeding control at the cost of a greater perioperative burden and longer recovery [33]. At the same time, Niaraees Zavare et al. showed that economic evaluations of HMB treatments remain heterogeneous and that LNG-IUS frequently represents a favorable cost-effective strategy despite differences in clinical effectiveness among treatment modalities [34]. Current treatment frameworks generally position LNG-IUS as an important minimally invasive option for appropriately selected women with HMB without major intracavitary pathology, while endometrial ablation/resection represents a uterus-preserving procedural alternative for women who have completed childbearing [35]. Therefore, the lower observed rates of hysterectomy and bleeding-related symptoms, together with higher satisfaction and more favorable adjusted FSFI outcomes following ER in our cohort, should not be generalized to all women with HMB. LNG-IUS remains reversible, less invasive, and clinically appropriate for many women, whereas ER requires operative intervention and is intended for women who have completed childbearing. Shared decision-making should therefore incorporate bleeding severity and etiology, reproductive preferences, expected bleeding patterns, tolerance of hormonal treatment, willingness to undergo an invasive procedure, and the potential need for subsequent treatment escalation.

The present study has several strengths. It evaluates two commonly used uterus-preserving treatment pathways within the same clinically defined real-world cohort and incorporates outcomes extending beyond short-term bleeding control, including subsequent hysterectomy, hysterectomy-free survival, treatment persistence, device discontinuation, subsequent procedural reintervention, treatment satisfaction, and sexual function. The baseline bleeding burden was characterized using PBAC, hemoglobin, and serum ferritin, while AUB etiology was categorized according to PALM–COEIN. In addition, complementary statistical approaches were used to examine the principal outcomes, including baseline-adjusted linear regression, Firth penalized logistic regression for the relatively uncommon hysterectomy endpoint, stabilized IPTW with a formal assessment of covariate balance, Kaplan–Meier analysis, conventional Cox regression, and IPTW-weighted Cox sensitivity analysis. Firth penalization was used to reduce small-sample maximum-likelihood bias associated with the relatively sparse hysterectomy outcome. Although the principal associations were generally directionally consistent across these analyses, all analyses were based on the same observational cohort and should therefore be interpreted within the limitations of the non-randomized study design.

This study also has several limitations. First, its retrospective, single-center, and non-randomized design, together with preference-based treatment allocation, introduces the possibility of selection bias and residual confounding. Although IPTW achieved an adequate balance across the measured baseline covariates included in the propensity model, unmeasured confounding cannot be excluded. In particular, several potentially relevant determinants of treatment choice were not uniformly available in the retrospective records and therefore could not be incorporated into the propensity-score model, leaving the possibility of residual confounding by indication. Furthermore, because hysterectomy was based on individualized clinical assessments and patient preferences rather than a prespecified objective threshold, variation in the decision to proceed to surgery may have influenced the primary outcome. Outcome ascertainment was based on a retrospective review of routine clinical records and was not performed by blinded or independent assessors. Accordingly, ascertainment bias cannot be excluded, particularly for patient-reported outcomes and outcomes involving clinical judgment, including the decision to proceed to hysterectomy. Second, treatment satisfaction was recorded as a binary clinical outcome rather than using a validated satisfaction instrument, and bleeding-related symptoms at follow-up were derived from routine clinical documentation rather than a prospectively standardized longitudinal bleeding assessment. Although baseline PBAC measurements were available, serial PBAC values were not used to quantify the treatment response throughout follow-up. Third, sexual function was patient-reported and may have been influenced by psychosocial, relationship, hormonal, and expectation-related factors that were not captured in the dataset. Fourth, the uterine size or uterine volume was not recorded as a continuous quantitative variable in the retrospective dataset; therefore, the potential relationship between uterine dimensions and hemoglobin levels or anemia could not be evaluated. Fifth, although hysterectomy dates permitted Kaplan–Meier and Cox analyses, detailed temporal trajectories of bleeding symptoms and the exact timing of all intermediate device removals, crossovers, and non-hysterectomy reinterventions were not uniformly available. In addition, the relatively limited follow-up duration precludes conclusions regarding long-term treatment durability; therefore, the present findings should be interpreted as reflecting short- to mid-term outcomes. Finally, the relatively small number of hysterectomy events—24 overall and only 4 following ER—resulted in wide CIs around some adjusted estimates despite the use of Firth penalization and complementary survival models. The exclusion of structural uterine pathology additionally limits generalizability to the broader population of women with HMB.

## 5. Conclusions

In this retrospective real-world cohort of women with medically refractory HMB without structural uterine pathology, hysteroscopic ER was associated with fewer subsequent hysterectomies and bleeding-related symptoms, higher treatment satisfaction, and more favorable sexual-function outcomes than initial treatment with the 52 mg LNG-IUS. Although the direction of the principal associations was generally consistent across the adjusted and sensitivity analyses, all analyses were derived from the same observational cohort and remain subject to residual confounding. A substantial proportion of women initially treated with LNG-IUS also experienced device discontinuation/removal or expulsion and subsequent procedural reintervention. Nevertheless, because treatment allocation was preference-based and non-randomized, the findings should not be interpreted as proof of causal treatment superiority. Rather, they provide real-world comparative evidence that may assist individualized counseling regarding expected short- to mid-term outcomes, bleeding control, sexual-function outcomes, treatment acceptability, and the potential need for subsequent intervention.

## Figures and Tables

**Figure 1:**
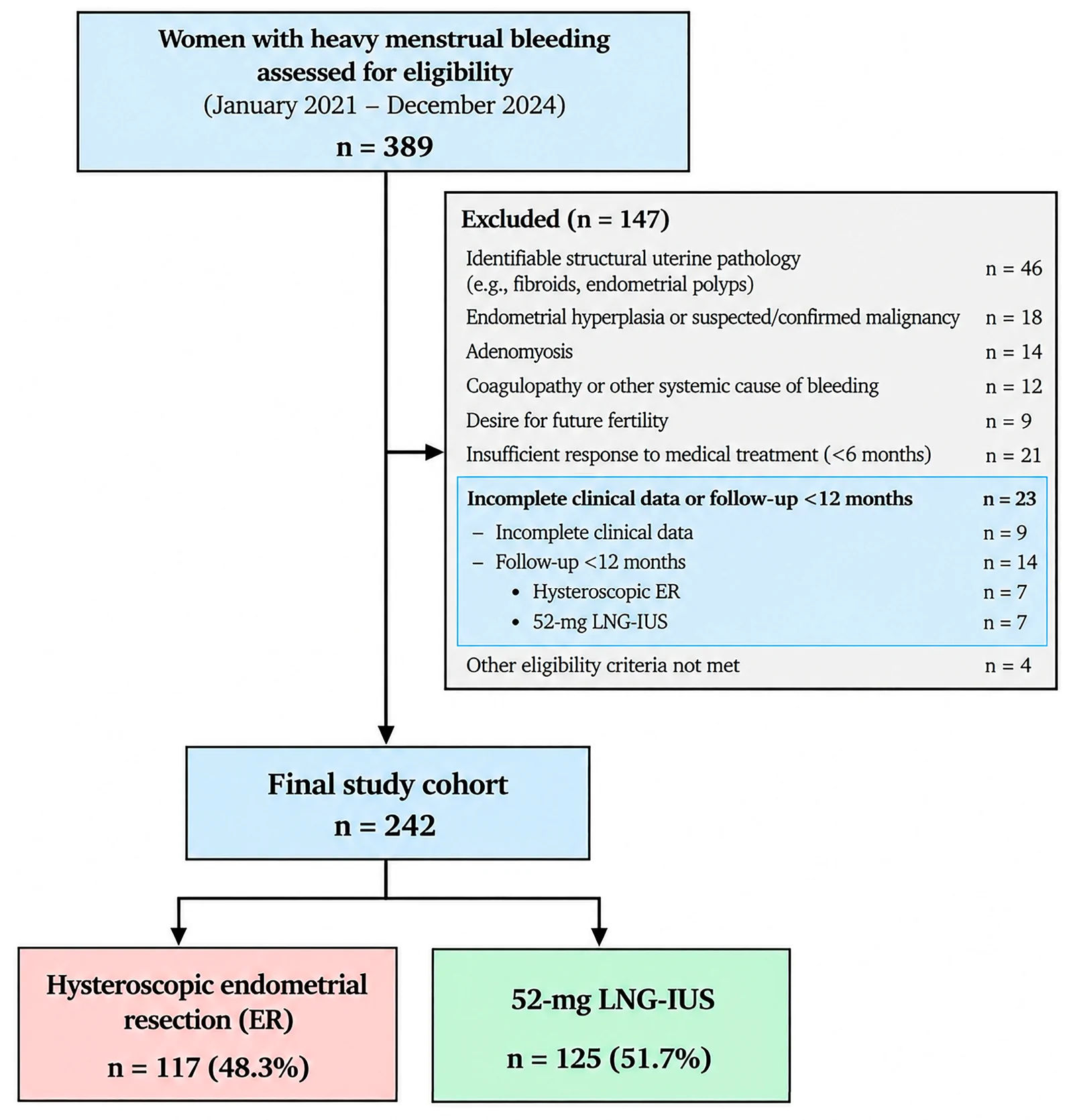
Patient selection flowchart. Flow diagram showing the identification, eligibility assessment, reasons for exclusion, and final treatment-group distribution of women with HMB included in the retrospective cohort.

**Figure 2:**
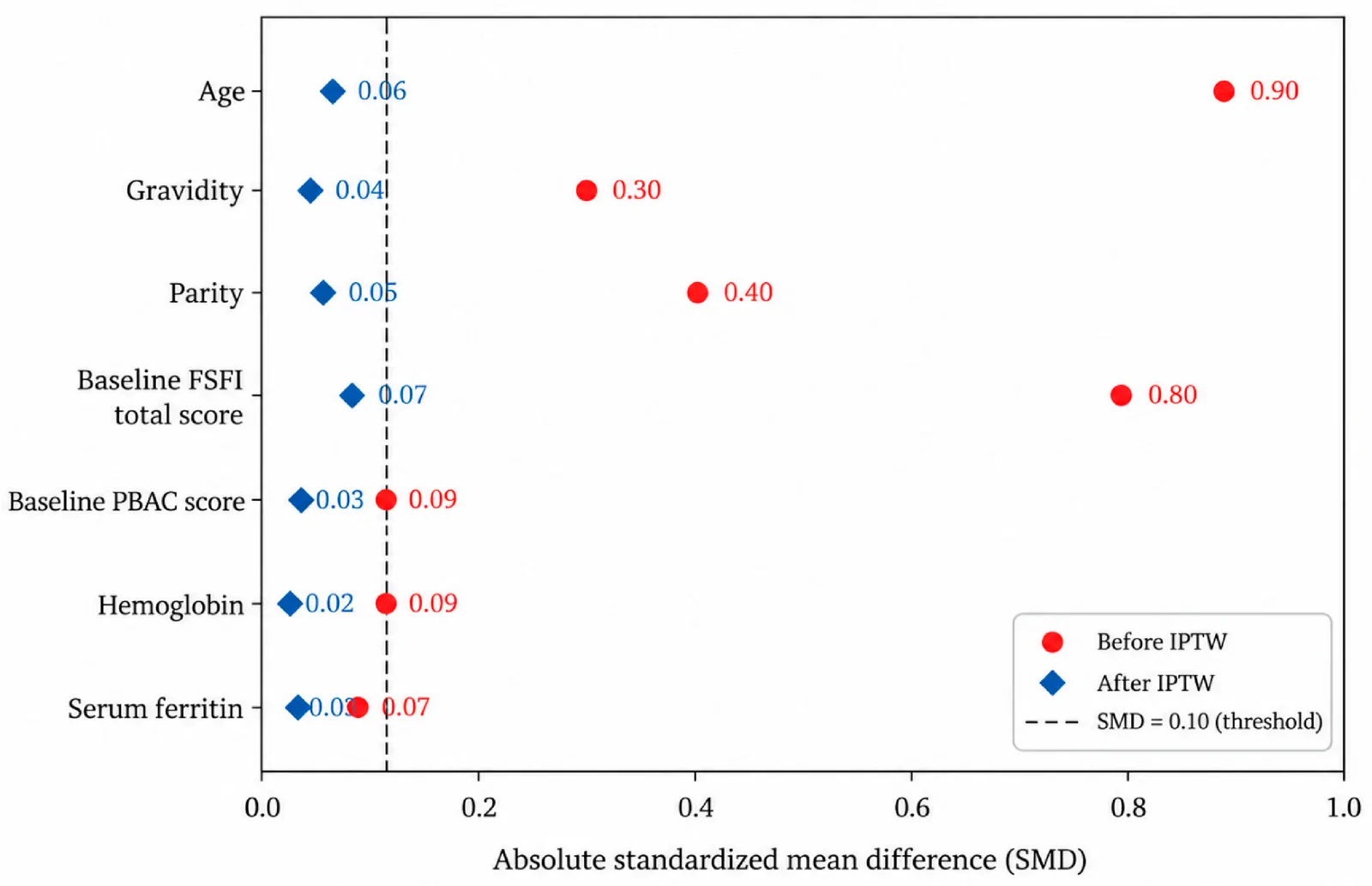
Covariate balance before and after IPTW. Absolute standardized mean differences (SMDs) are shown for the prespecified baseline covariates before and after the application of stabilized inverse probability of treatment weights (IPTWs). The vertical dashed line at an absolute SMD of 0.10 represents the threshold for adequate covariate balance. After weighting, all included baseline covariates demonstrated absolute SMDs < 0.10.

**Figure 3:**
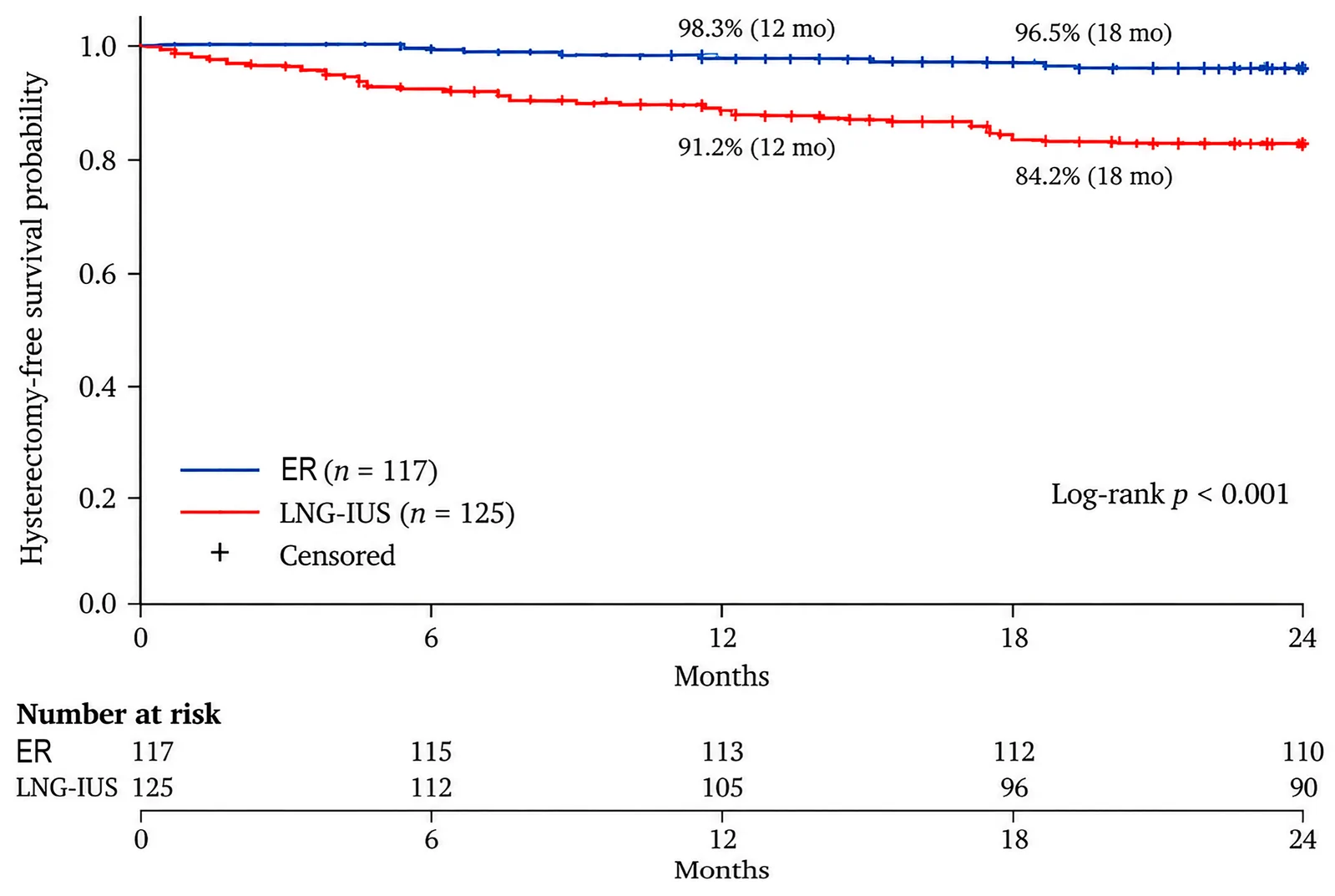
Kaplan–Meier curves for hysterectomy-free survival according to treatment group. Kaplan–Meier curves illustrate hysterectomy-free survival following ER and initial treatment with the 52 mg levonorgestrel-releasing intrauterine system (LNG-IUS). Women who did not undergo hysterectomy were censored at their last documented follow-up. Hysterectomy-free survival differed significantly between treatment groups (log-rank *p* < 0.001). Numbers at risk are displayed below the survival curves.

**Table 1:** Baseline and follow-up characteristics according to treatment group.

Variable	ER (*n* = 117)	LNG-IUS (*n* = 125)	Absolute SMD	*p* Value
Age, years	43.4 ± 3.0	39.9 ± 4.7	0.90	<0.001 ***
Gravidity	3.0 [2.0–4.0]	3.0 [2.0–4.0]	0.30	0.029
Parity	2.0 [2.0–3.0]	2.0 [1.0–3.0]	0.40	0.012
Vaginal deliveries	1.0 [0.0–2.0]	1.0 [0.0–2.0]	0.20	0.396
Baseline PBAC score	286 [244–338]	278 [238–329]	0.09	0.472
Hemoglobin, g/dL	11.1 ± 1.2	11.2 ± 1.1	0.09	0.503
Serum ferritin, ng/mL	17.8 [10.6–27.4]	18.6 [11.2–28.1]	0.07	0.581
Baseline FSFI total score	27.2 ± 2.3	28.8 ± 2.1	0.80	<0.001 ***
Hemoglobin at 12 months, g/dL	13.2 ± 0.9	12.5 ± 1.1	—	<0.001 ***
Change in hemoglobin from baseline, g/dL	+2.1 ± 1.1	+1.3 ± 1.2	—	<0.001 ***
Follow-up duration, months	16.8 ± 3.7	16.2 ± 3.5	0.20	0.197

Data are presented as the mean ± SD or median [IQR], as appropriate. The change in hemoglobin was calculated as the 12-month value minus the baseline value; positive values indicate an increase. SMD = standardized mean difference; PBAC = Pictorial Blood Assessment Chart; FSFI = Female Sexual Function Index; LNG-IUS = levonorgestrel-releasing intrauterine system; ER = hysteroscopic endometrial resection. Welch’s independent-samples *t* test was used for approximately normally distributed continuous variables, and the Mann–Whitney U test was used for non-normally distributed or ordinal/count variables. Absolute SMDs were reported for baseline characteristics to quantify the between-group imbalance; an absolute SMD < 0.10 was considered indicative of an adequate balance. SMDs are descriptive measures and were not subjected to significance testing. *** *p* < 0.001.

**Table 2:** Distribution of abnormal uterine bleeding etiologies according to the PALM–COEIN classification.

PALM–COEIN Category	Overall (*n* = 242)	ER (*n* = 117)	LNG-IUS (*n* = 125)
AUB-O—Ovulatory dysfunction	69 (28.5)	32 (27.4)	37 (29.6)
AUB-E—Endometrial	157 (64.9)	77 (65.8)	80 (64.0)
AUB-N—Not otherwise classified	16 (6.6)	8 (6.8)	8 (6.4)
Overall distribution, *p* value †	—	0.925	—

Data are presented as *n* (%). AUB etiologies were categorized according to the FIGO PALM–COEIN classification using the predominant baseline etiologic category. Only the non-structural AUB categories represented in the study cohort (AUB-O, AUB-E, and AUB-N) are shown; categories excluded by the study eligibility criteria or not represented in the cohort were omitted from the table. † The overall distribution of AUB-O, AUB-E, and AUB-N between the ER and LNG-IUS groups was compared using the chi-square test. AUB, abnormal uterine bleeding; O, ovulatory dysfunction; E, endometrial; N, not otherwise classified; ER, hysteroscopic endometrial resection; LNG-IUS, 52 mg levonorgestrel-releasing intrauterine system.

**Table 3:** Clinical outcomes according to treatment group.

Outcome	ER (*n* = 117)	LNG-IUS (*n* = 125)	Effect Estimate (95% CI)	*p* Value
Hysterectomy during follow-up	4 (3.4)	20 (16.0)	RR 4.7 (1.6–13.3)	0.001 ***
Any bleeding-related symptom	4 (3.4)	57 (45.6)	RR 13.3 (5.0–35.6)	<0.001 ***
Intermenstrual spotting	0 (0.0)	37 (29.6)	RD 29.6% (21.6–37.6)	<0.001 ***
Treatment satisfaction †	110 (94.0)	55 (44.0)	RR 0.47 (0.4–0.6)	<0.001 ***

Data are presented as *n* (%). RR, risk ratio; RD, risk difference; CI, confidence interval; LNG-IUS, 52 mg levonorgestrel-releasing intrauterine system; ER, hysteroscopic endometrial resection. Effect estimates compare LNG-IUS with ER. Fisher’s exact test was used for categorical comparisons. Because no intermenstrual spotting events occurred in the ER group, the absolute risk difference was reported instead of a risk ratio for this outcome. † Treatment satisfaction was recorded as a binary clinical outcome. *** *p* ≤ 0.001.

**Table 4:** Change in FSFI total score from baseline to 12 months.

FSFI Total Score	ER (*n* = 117)	LNG-IUS (*n* = 125)	Between-Group Difference (95% CI)	*p* Value
Baseline	27.2 ± 2.3	28.8 ± 2.1	—	—
12 months	29.2 ± 2.3	28.6 ± 2.3	—	—
Change from baseline (ΔFSFI)	2.0 ± 1.0	−0.2 ± 2.2	2.2 (1.7–2.6)	<0.001 ***
Within-group *p* value	<0.001	0.361	—	—
Cohen’s *d* for between-group difference in change	—	—	1.2	—

Data are presented as the mean ± SD unless otherwise indicated. The change (ΔFSFI) was calculated as the 12-month FSFI total score minus the baseline FSFI total score; therefore, positive values indicate an improvement. The between-group difference in the change was calculated as ER minus LNG-IUS. Within-group *p* values represent paired comparisons between baseline and 12-month FSFI total scores, whereas the between-group *p* value represents the comparison of change scores between the ER and LNG-IUS groups. Cohen’s *d* represents the standardized effect size for the between-group difference in the change. FSFI, Female Sexual Function Index; CI, confidence interval; LNG-IUS, 52 mg levonorgestrel-releasing intrauterine system; ER, hysteroscopic endometrial resection. *** *p* < 0.001.

**Table 5:** Baseline-adjusted robust linear regression for 12-month FSFI total score.

Predictor	Adjusted B	95% CI	*p* Value
LNG-IUS vs. ER	−2.2	−2.6 to −1.7	<0.001 ***
Baseline FSFI total score, per 1-point increase	0.5	0.4 to 0.6	<0.001 ***
Age, per 1-year increase	−0.2	−0.3 to −0.1	<0.001 ***
Parity, per 1-birth increase	0.0	−0.1 to 0.2	0.711

The dependent variable was the 12-month FSFI total score. Regression coefficients (B) are unstandardized regression coefficients obtained from a multivariable linear regression model using heteroskedasticity-consistent HC3 robust standard errors. ER was the reference treatment group. A negative coefficient for LNG-IUS therefore indicates a lower adjusted 12-month FSFI total score compared with ER. The model was adjusted for the baseline FSFI total score, age, and parity. FSFI, Female Sexual Function Index; CI, confidence interval; LNG-IUS, 52 mg levonorgestrel-releasing intrauterine system; ER, hysteroscopic endometrial resection. *** *p* < 0.001.

**Table 6:** Propensity-score/IPTW sensitivity analysis.

Outcome/Covariate	Unweighted Estimate/Absolute SMD	IPTW-Weighted Estimate/Absolute SMD	95% CI	*p* Value
Covariate balance				
Age	0.90	0.06	—	—
Gravidity	0.30	0.04	—	—
Parity	0.40	0.05	—	—
Baseline FSFI total score	0.80	0.07	—	—
Baseline PBAC score	0.09	0.03	—	—
Hemoglobin	0.09	0.02	—	—
Serum ferritin	0.07	0.03	—	—
Clinical outcomes, LNG-IUS vs. ER				
Hysterectomy	RR 4.7	RR 8.9	2.8–28.4	<0.001 ***
Any bleeding-related symptom	RR 13.3	RR 12.8	5.0–32.9	<0.001 ***
Treatment satisfaction	RR 0.47	RR 0.48	0.39–0.59	<0.001 ***
12-month FSFI total score	B −2.2	B −2.1	−2.5 to −1.6	<0.001 ***

Propensity scores were estimated using age, gravidity, parity, the baseline FSFI total score, the baseline PBAC score, hemoglobin, and serum ferritin. Stabilized inverse probability of treatment weights (IPTWs) were applied. The covariate balance before and after weighting was assessed using absolute standardized mean differences (SMDs); an absolute SMD < 0.10 was considered indicative of an adequate covariate balance. SMDs are descriptive measures of the covariate balance and were not subjected to significance testing. Weighted outcome models used robust standard errors. Risk ratios (RRs) compare LNG-IUS with ER. For the 12-month FSFI total score, B represents the unstandardized weighted regression coefficient for LNG-IUS relative to ER; negative values indicate lower FSFI scores with LNG-IUS. CI, confidence interval; FSFI, Female Sexual Function Index; PBAC, Pictorial Blood Assessment Chart; LNG-IUS, 52 mg levonorgestrel-releasing intrauterine system; ER, hysteroscopic endometrial resection. *** *p* < 0.001.

**Table 7:** Time-to-hysterectomy analysis according to treatment group.

Outcome	ER (*n* = 117)	LNG-IUS (*n* = 125)	Effect Estimate (95% CI)	*p* Value
Hysterectomy during follow-up, *n* (%)	4 (3.4)	20 (16.0)	—	—
Median time to hysterectomy among women with an event, months [IQR]	14.3 [11.8–16.6]	10.8 [7.6–13.7]	—	—
12-month hysterectomy-free survival, %	98.3	91.2	—	—
18-month hysterectomy-free survival, %	96.5	84.2	—	<0.001 †***
Unadjusted Cox model, LNG-IUS vs. ER	—	—	HR 5.2 (1.8–15.2)	0.003
Age- and parity-adjusted Cox model, LNG-IUS vs. ER	—	—	HR 11.6 (3.4–39.4)	<0.001 ***
IPTW-weighted Cox model, LNG-IUS vs. ER	—	—	HR 9.8 (3.0–32.1)	<0.001 ***

Time to hysterectomy was calculated from the index treatment to the date of hysterectomy; women who did not undergo hysterectomy were censored at their last documented follow-up. Hysterectomy-free survival was estimated using the Kaplan–Meier method. † The corresponding *p* value represents the overall comparison of Kaplan–Meier survival curves using the log-rank test. Cox proportional-hazards models were used to estimate hazard ratios (HRs), with ER as the reference group. The adjusted Cox model included age and parity. The IPTW-weighted Cox model used the stabilized inverse probability of treatment weights with robust standard errors. The proportional-hazards assumption was assessed using Schoenfeld residuals. HR, hazard ratio; CI, confidence interval; IPTW, inverse probability of treatment weighting; LNG-IUS, 52 mg levonorgestrel-releasing intrauterine system; ER, hysteroscopic endometrial resection. *** *p* < 0.001.

**Table 8:** Univariable and multivariable Firth penalized logistic regression analysis for hysterectomy.

Predictor	Univariable OR (95% CI)	*p* Value	Adjusted OR (95% CI)	*p* Value
LNG-IUS vs. ER	5.38 (1.85–15.66)	0.002	18.9 (5.0–71.9)	<0.001 ***
Age, per 1-year increase	1.18 (1.07–1.30)	0.001	1.5 (1.3–1.7)	<0.001 ***
Parity, per 1-birth increase	1.13 (0.84–1.53)	0.419	1.1 (0.8–1.5)	0.661

Firth penalized logistic regression was used because of the relatively small number of hysterectomy events. Hysterectomy during follow-up was the dependent variable. ER was the reference treatment group. The multivariable model simultaneously included the treatment group, age, and parity. OR, odds ratio; CI, confidence interval; LNG-IUS, 52 mg levonorgestrel-releasing intrauterine system; ER, hysteroscopic endometrial resection. *** *p* < 0.001.

**Table 9:** LNG-IUS removal, expulsion, and subsequent reintervention.

Outcome	LNG-IUS (*n* = 125), *n* (%)
Device discontinuation/removal or expulsion	49 (39.2)
Persistent or recurrent HMB	24 (19.2)
Persistent intermenstrual spotting/bleeding intolerance	11 (8.8)
Spontaneous complete or partial expulsion	8 (6.4)
Pain, cramping, or other device-related intolerance	6 (4.8)
Subsequent procedural reintervention	32 (25.6)
Hysterectomy	20 (16.0)
Subsequent ER	8 (6.4)
LNG-IUS replacement/reinsertion	4 (3.2)

Data are presented as *n* (%), with percentages calculated using the 125 women initially treated with LNG-IUS as the denominator. Device discontinuation/removal or expulsion and subsequent procedural reintervention were evaluated as separate outcomes and should not be summed. HMB, heavy menstrual bleeding; LNG-IUS, 52 mg levonorgestrel-releasing intrauterine system.

## Data Availability

The datasets generated and analyzed during the current study are available from the corresponding author upon reasonable request.

## Abbreviations

AUB	Abnormal Uterine Bleeding
AUB-O	Ovulatory Dysfunction
AUB-E	Endometrial
AUB-N	Not Otherwise Classified
CI	Confidence Interval
ER	Hysteroscopic Endometrial Resection
FIGO	International Federation of Gynecology and Obstetrics
FSFI	Female Sexual Function Index
HMB	Heavy Menstrual Bleeding
HR	Hazard Ratio
IPTW	Inverse Probability of Treatment Weighting
IQR	Interquartile Range
LNG-IUS	Levonorgestrel-Releasing Intrauterine System
OR	Odds Ratio
PBAC	Pictorial Blood Assessment Chart
RD	Risk Difference
RR	Risk Ratio
SD	Standard Deviation
SMD	Standardized Mean Difference
